# No Evidence for Cardiac Dysfunction in Kif6 Mutant Mice

**DOI:** 10.1371/journal.pone.0054636

**Published:** 2013-01-23

**Authors:** Abdul Hameed, Ellen Bennett, Barbara Ciani, Loes P. C. Hoebers, Roy Milner, Allan Lawrie, Sheila E. Francis, Andrew J. Grierson

**Affiliations:** 1 Department of Cardiovascular Science, Medical School, University of Sheffield, Sheffield, United Kingdom; 2 Sheffield Institute for Translational Neuroscience, University of Sheffield, Sheffield, United Kingdom; 3 Department of Chemistry, University of Sheffield, Sheffield, United Kingdom; University of Tampere, Finland

## Abstract

A *KIF6* variant in man has been reported to be associated with adverse cardiovascular outcomes after myocardial infarction. No clear biological or physiological data exist for Kif6. We sought to investigate the impact of a deleterious *KIF6* mutation on cardiac function in mice. Kif6 mutant mice were generated and verified. Cardiac function was assessed by serial echocardiography at baseline, after ageing and after exercise. Lipid levels were also measured. No discernable adverse lipid or cardiac phenotype was detected in Kif6 mutant mice. These data suggest that dysfunction of Kif6 is linked to other more complex biological/biochemical parameters or is unlikely to be of material consequence in cardiac function.

## Introduction

There has been considerable debate within the cardiovascular community regarding the role of kinesin-like protein 6 (Kif6) in coronary artery disease. Initial reports using a candidate gene based approach in several atherosclerosis population cohorts observed an increase risk of adverse coronary events in carriers of the rs20455 C variant in the *KIF6* gene which leads to a Trp719Arg substitution in the Kif6 protein [1], [2], [3], [4]. These findings were controversial and refuted by a large GWAS meta-analysis using 19 case-control studies [5] where cases were defined either by a history of prior myocardial infarction and/or the presence of coronary artery disease at angiography. Furthermore no association between the kif6 variant and coronary events was observed in the most recent large meta-analysis [6]. However, evidence from interventional randomized controlled trials (RCTs) with statins associated carriage of this genotype with a greater clinical response to statin therapy [7], [8], [9], [10]. As a clinical test based on *KIF6* genotype was already on the market, the utility and clinical significance of this was in doubt [11], [12]. More recently, retrospective analyses from several large independent RCTs [13], [14], [15], [16], [17] have not observed an increased risk of adverse vascular events or an attenuated response to statin therapy amongst Kif6 719Arg carriers. In an attempt to reconcile these discrepant findings, Ference et al hypothesized that the rs20455 C variant in the *KIF6* gene may influence LDL levels. By performing a regression meta-analysis involving almost 145,000 patients, they reported that carriers demonstrated greater reductions in LDL, when treated with statins and consequently greater reduction in clinical events when compared to non-carriers [6].

Kif6 is a member of the Kinesin 9 superfamily but the precise molecular function of Kif6 is not known. It is likely to play a role in the cellular transport of proteins along microtubules [18], [19] and could plausibly be involved in cellular transport in the cardiovascular system. Hitherto, all studies have focused on investigating polymorphism in the *KIF6* gene within the context of coronary artery disease yet to our knowledge there has been no investigation (experimental or clinical) into its function (if any) on heart function. We hypothesised that if loss of Kif6 function was associated with cardiovascular disease, then mice with a deleterious mutation in the Kif6 motor domain may exhibit defects in cardiac physiology.

Therefore, we identified mice with an N-ethyl-N-nitrosourea (ENU) induced mutation in the Kif6 motor, and investigated the structural and functional cardiac phenotypes by high frequency transthoracic echocardiography.

## Methods

Kif6 mutant mice were obtained from the RIKEN Bioresource Centre (Japan, RBRC03194), backcrossed 5 generations to C57BL/6 and classed as incipient congenics. The likelihood that a phenotype would arise because of a confounding mutation in the RIKEN library is statistically slim (p<0.002) [20].

RT-PCR and Western blot analysis of Kif6 expression was performed according to standard methods [21]. A rabbit polyclonal antibody (Kif6 C165) was generated using a peptide containing 20 C-terminal amino acids of human Kif6 as an immunogen. Specificity of the antibody was demonstrated by showing competition of the antisera with the immunizing peptide in cells transfected with recombinant full length human Kif6 (data not shown).

Lipid levels were assessed at 18 weeks of age. Cardiac function was measured using serial echocardiography in mice (some were exercised on running wheels) for periods of time up to 43 weeks. The supplemental methods and data contain additional information. All animal work was carried out in strict accordance with UK Home Office regulations under project licence approval 40/3307. The experimental protocols were approved by the University of Sheffield ethical review board. All echocardiography was carried out under isoflurane anaesthesia and all efforts were made to minimize suffering.

## Results

### Kif6 Expression in Mouse Tissues, Generation of the kif6 Mutation in Mice, Confirmation and Prediction of Effect on the Motor Domain

**Figure 1:**
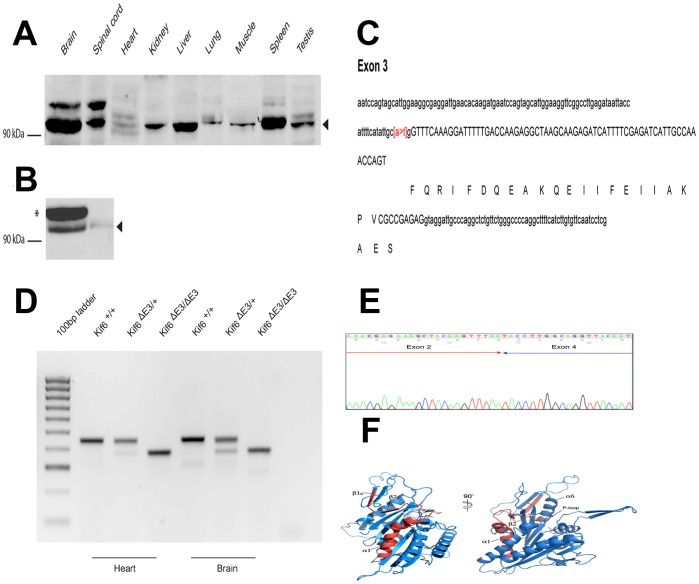
Kif6 expression in tissues, cells, mutant mice and structural information. (A) Multi-tissue Western blot analysis of Kif6 protein expression. 20 µg of total protein from each tissue was separated by PAGE. The predicted molecular weight of Kif6 is 92KDa. An arrowhead indicates the Kif6 protein recognized by the C165 antisera. (B) Transfected c-myc epitope tagged Kif6a in HEK 293 cells and Kif6 in primary endothelial cells (HUVEC). (C) DNA sequence of exon 3 in Kif6 mutants, showing the A>T mutation at the −2 position of the exon 3 splice acceptor site. (D) RT-PCR of Kif6 RNA extracted from heart and brain tissue of wild type and mutant mice. (E) cDNA sequence of the mutant RT-PCR product, showing exon 2 reading into exon 4. (F) Ribbon representation of mouse KIF6 motor domain modeled on the crystal structure of the human KIF9 motor domain in complex with ADP (PDB 3nwn). The ADP molecule is represented in a sticks model and the region (residue K59 to residue S86) corresponding to exon 3 deletion is shown in red. Secondary structure elements are numbered according to the convention for the kinesin motors.

Kif6 protein was detected in a variety of tissues including heart and endothelial cells (Figure 1A and B). To generate a model of *KIF6* mutation in mice, exons 2, 3 and 7 were sequenced in >7000 DNA samples from ENU-mutagenised mice in the RIKEN Biorepository. This led to the identification of an A to G mutation in the exon 3 splice acceptor site of the Rgsc2221 (RBRC03194) mouse line (Fig. 1C). Since this nucleotide change is predicted to disrupt splicing of the *KIF6* mRNA, we re-derived these mice by microinjection of sperm from an F1 Rgsc2221 male into C57BL/6 oocytes so as to permit experimental testing of the possible role for Kif6 in cardiac dysfunction. Subsequently, offspring carrying the mutation were identified by PCR and HpyCH4V restriction digest, and maintained on the C57BL6/J background.

We extracted RNA from *KIF6* mutant and wild type littermates, and conducted RT-PCR with forward and reverse primers located in exons 1 and 5 respectively, to investigate any differences in splicing of the *KIF6* mRNA (Fig. 1D). This revealed the amplification of a shorter PCR product in heterozygous and homozygous mutant mice, implying an alteration in splicing. Sequencing of the shorter product revealed that the effect of the mutation is to cause exon 3 to be skipped from the *KIF6* mRNA (Fig. 1E). This leads to a novel transcript encoding a shortened Kif6 protein with a 25 amino acid in-frame deletion in the motor domain. On this basis we propose the name *KIF6* ^ΔE3^ for this allele. Kif6 shares 38% identical amino acid sequence with human Kif9. Kinesin motor domains all have rather similar structures, consisting of eight β-sheet strands sandwiched between six α-helices (three on either side, Fig. 1F). The core of the motor domain is made up by strand β1 up to and including helix α6. The major effect of exon 3 deletion from the *KIF6* gene, is to generate a product missing several elements of secondary structure necessary to support the core of the protein (deletion of β1c-β2-α1). In addition, the nucleotide-binding pocket lies in a groove partially formed by helix α1, which is absent in the Kif6 mutant protein. Helix α1 leads directly into the highly conserved N−1 region (nucleotide binding region or P-loop), and contains conserved residues across kinesins and myosins. The P-loop is the highest conserved region across kinesin and myosin families, therefore preceding structural elements such as helix α1, are crucial to maintain the loop conformation necessary for motor function even though they do not seem to bear any specific function. It is therefore highly likely that the gene produced by deletion of exon 3 has a misfolded motor domain, unable to bind or hydrolyze ATP.

### Lipid and Cardiac Phenotype of Kif6 Mutant Mice

**Figure 2:**
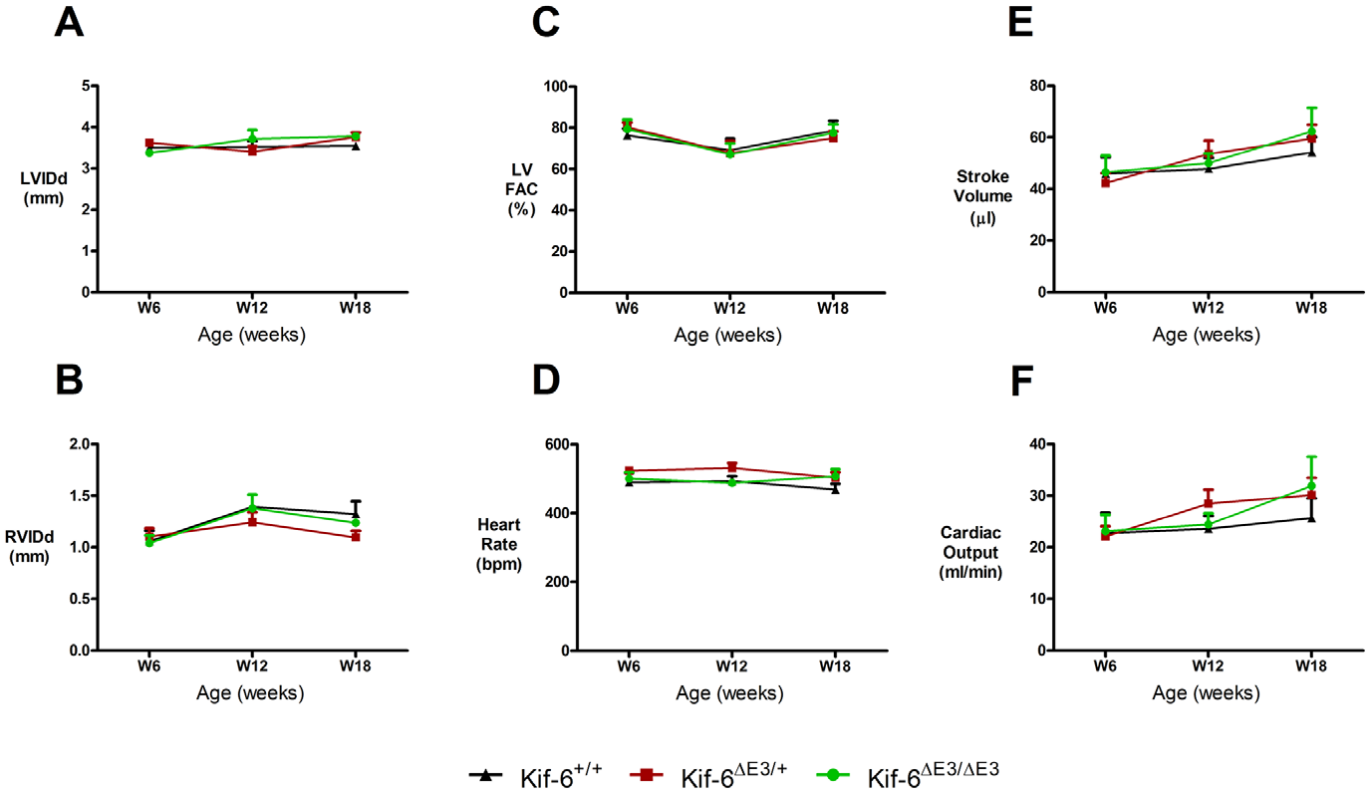
Serial cardiac function in adult male Kif6 mutant mice. Serial assessment (6–18 weeks) revealed no significant difference at either time point in size of A) Left or B) Right ventricle in diastole, C) LV contractility measured by fractional area change (FAC), D) heart rate, E) Stroke volume or F) Cardiac output. N = 4 per group.

Fasted lipid levels at 18 weeks of age indicated trends toward higher triglyceride levels in mutant mice but this did not reach statistical significance (Figure S1). Echocardiography at 6 weeks of age revealed no significant differences in Left (LVIDd), right (RVIDd) ventricle cavity size (Fig. 2a and b) or LV contractility (Fig. 2c) and tissue Doppler velocities (Table S1). When contractility was defined with M-Mode derived indices, *KIF6* ^ΔE3/+^ mice had mildly reduced LV fractional shortening and ejection fractions compared to homozygous mice only (Table S1). Left ventricle derived heart rate (Fig. 2d), stroke volume (µl) (Fig. 2e), aortic VTI (cm) and cardiac output (Fig. 2f) were not significantly different between groups (Table S1).

To evaluate cardiac function with increasing age, serial echocardiographic studies in mice after a further 6 and 12 weeks were performed (Table S1). No significant differences between the three strains of mice in all of the twelve parameters studied were found, except for a higher LVPWTDISa velocity in the *KIF6* ^ΔE3/+^ mice at 18 weeks of age (Table S1). Echocardiography was also performed in older mice at 43 weeks of age and we observed no evidence of reduced cardiac function in *KIF6* ^ΔE3/ΔE3^ homozygous mice (Table S1).

### Exercise Capacity and Cardiac Function in Adult Mice

To determine if a physiological stressor such as exercise could lead to the development of an abnormal cardiac phenotype, voluntary wheel running was assessed in female *KIF6* ^ΔE3/ΔE3^ mice after an exercise period of 13 weeks.

There were no significant differences in the distances (>5 Km/night) run by the mice (data not shown) or the relevant ventricular physiology parameters (Figure S2 and Table S2).

## Discussion

This study is the first to undertake whole animal research with mutant *KIF6* mice, and the first to attempt to find a physiological cardiac effect related to altered Kif6 function. Only one other study has attempted to link *KIF6* genotype with a vascular biological process, namely endothelial progenitor cell (EPC) production in humans. This study revealed that the Arg/Arg *KIF6* genotype was associated with a lower tendency to produce late outgrowth EPCs [22]. This was suggested to be as a result of Kif6 effects on cytokinesis.

Given the existing expression data detailing high Kif6 mRNA expression in rodent cardiac muscle (http://www.ebi.ac.uk/gxa/gene/ENSRNOG00000011453?ef=organism_part) which we formally confirm in mouse heart in this study, we speculated that Kif6 dysfunction might impact lipid profiles, cardiac structure and function at rest or after exercise in mice, despite there being no prior human association studies. Our data also clearly show that there is no effect of disrupting the Kif6 motor domain on cardiac structure or function even when mice are studied up to the age of 43 weeks or under exercise conditions. There was no significant impact of the Kif6 mutation upon lipid levels; we assert therefore, that it is unlikely that dysfunction of the Kif6 motor protein has any discernable effect on cardiac function. Interestingly, and in support of our work, Davani *et al*. [22] did not observe any significant difference in LV ejection fraction after acute myocardial infarction in relation to *KIF6* genotype.

### Study Limitations

In our study we investigated the effects of a mutation that lies within the motor domain of the *KIF6* gene, whereas the variant studied in humans lies within the tail domain. However given that the motor domain mutation under investigation here is likely to lead a non-functional *KIF6* gene, we believe nonetheless that it remains a useful model to determine the role of kif6 in cardiac function.

Our study primarily investigated whether mice with non-functioning kif6 had impaired ventricular function. Although we did not specifically investigate atherosclerosis, we observed no significant differences in lipid levels in *KIF6* ^ΔE3/ΔE3^ mice. Group sizes were 8 animals and study power was 74% for these measures. It is possible that much larger group sizes may have revealed statistically significant differences. However, unlike humans, mice have much lower LDL levels and given the reported link between the kif6 variant with higher LDL levels and adverse coronary events [6], it is possible that effects of Kif6 may only be revealed on a proatherogenic mouse background (such as apoE or LDL-receptor null mice fed a high fat diet).

### Conclusion

The data presented here strongly suggest that mice with a mutation within the motor domain of Kif6 (*KIF6* ^ΔE3^) mutant mice have no discernible adverse cardiac phenotype.

## Supporting Information

Figure S1 Lipid levels in Kif6 mutant mice at 18 weeks of age. No significant differences were obtained, n = 6−8 each group.
(TIF)

Figure S2 Exercise and cardiac function in adult female Kif6 mutant mice. After voluntary wheel running for 13 weeks, no significant differences were observed between groups in A) Cavity size (diastole), B) LV mass, C) Fractional shortening (%), D Fractional area change (FAC), E) posterior wall systolic wave velocity using tissue Doppler, F) cardiac output, n = 4.
(TIF)

Table S1 Detailed physiological and echocardiographic dataset for adult male Kif6 mutant mice undergoing serial echocardiography between 6–43 weeks of age. Data are presented as mean [SEM].
(PDF)

Table S2 Detailed physiological and echocardiographic dataset for adult female Kif6 mutant mice after continuous voluntary wheel running for 13 weeks. Data are presented as mean [SEM].
(TIF)

Methods S1 Supplementary methods.
(DOCX)
