# Supplementary figures and images for: No Evidence for Cardiac Dysfunction in Kif6 Mutant Mice
Source: PLoS One. 2013 Jan 23;8(1):e54636. doi: 10.1371/journal.pone.0054636 (PMC3552957; doi:10.1371/journal.pone.0054636)

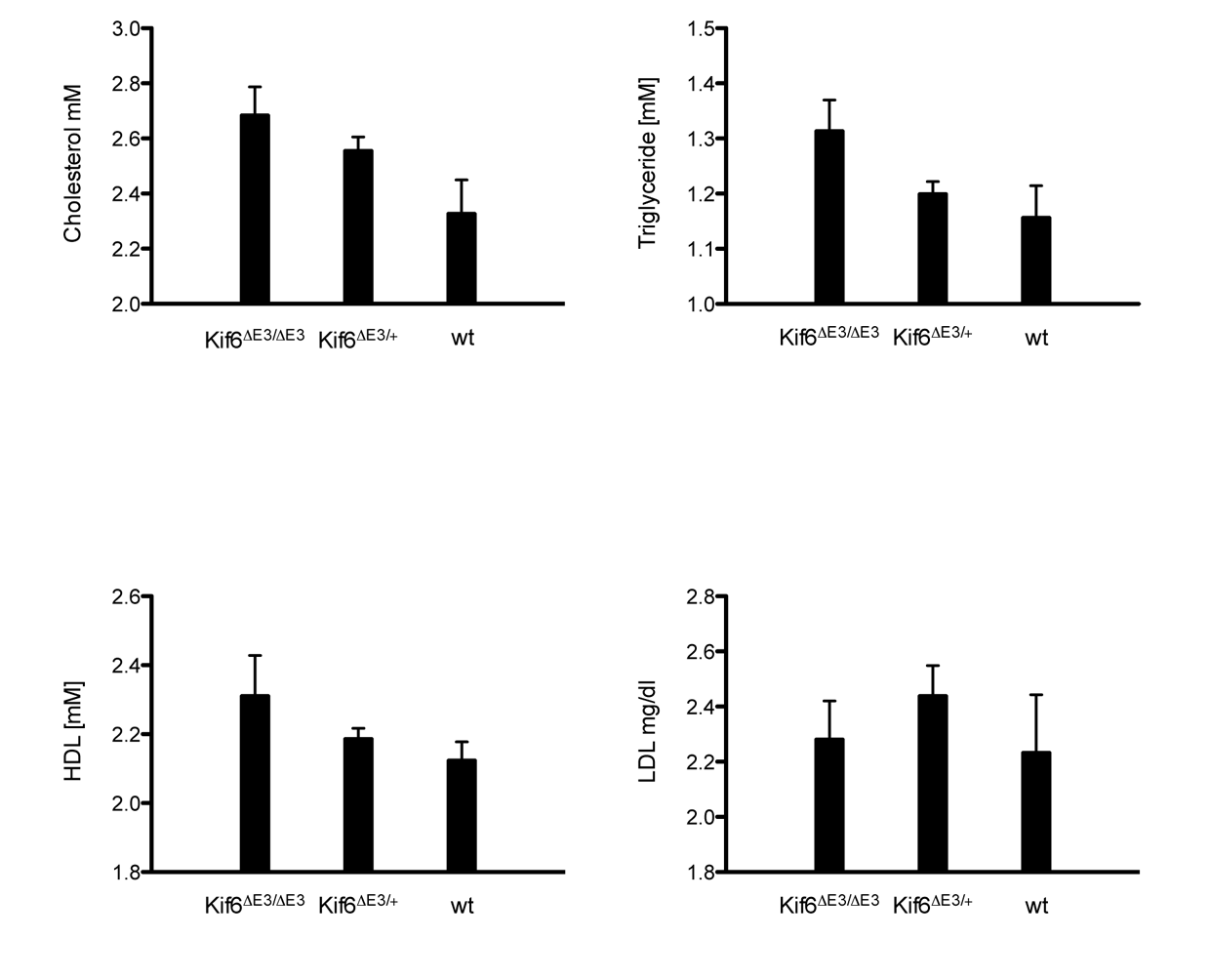

Supplement: Figure S1 — Lipid levels in Kif6 mutant mice at 18 weeks of age. No significant differences were obtained, n = 6−8 each group. (TIF) [file pone.0054636.s001.tif]

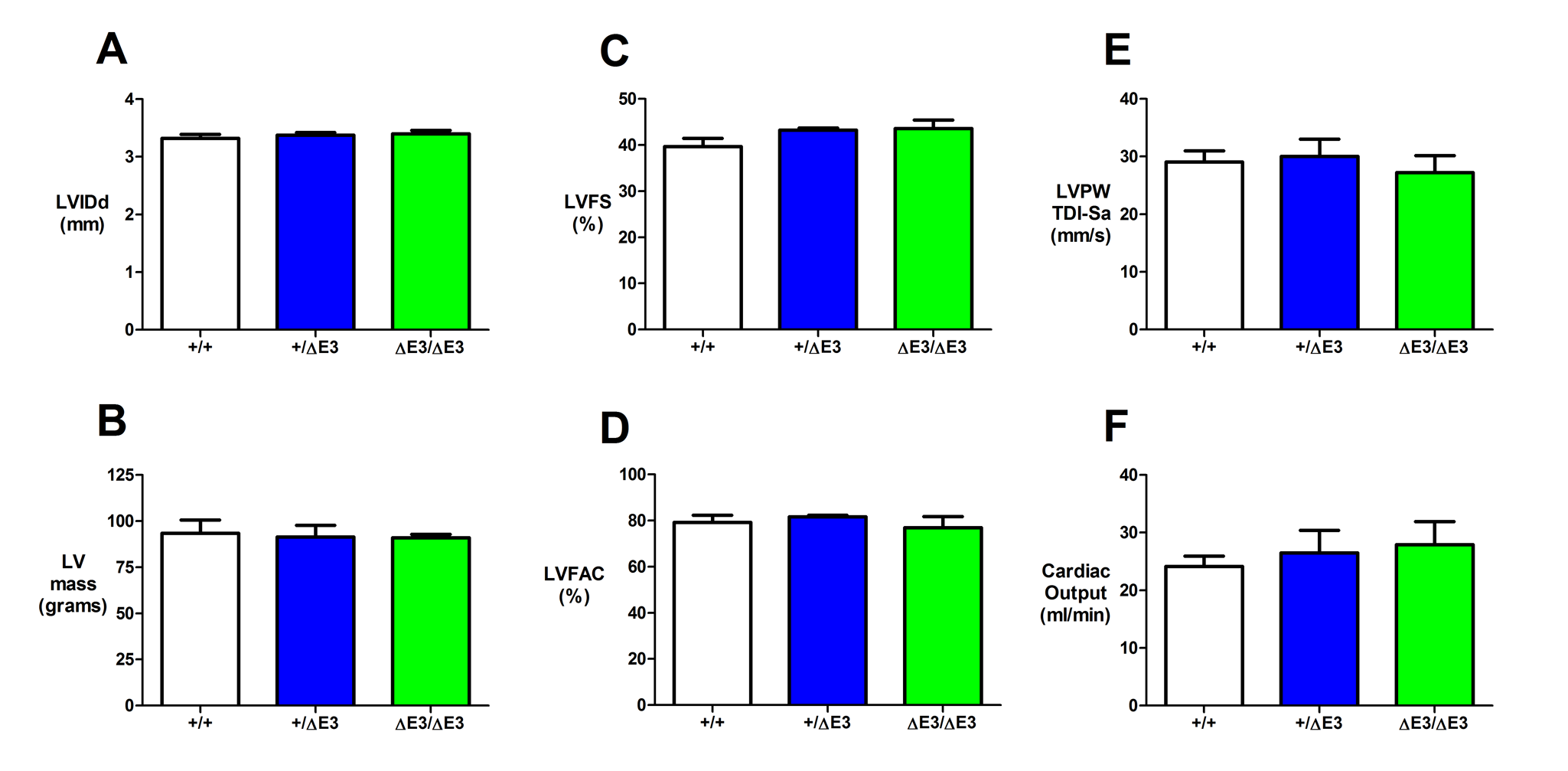

Supplement: Figure S2 — Exercise and cardiac function in adult female Kif6 mutant mice. After voluntary wheel running for 13 weeks, no significant differences were observed between groups in A) Cavity size (diastole), B) LV mass, C) Fractional shortening (%), D Fractional area change (FAC), E) posterior wall systolic wave velocity using tissue Doppler, F) cardiac output, n = 4. (TIF) [file pone.0054636.s002.tif]

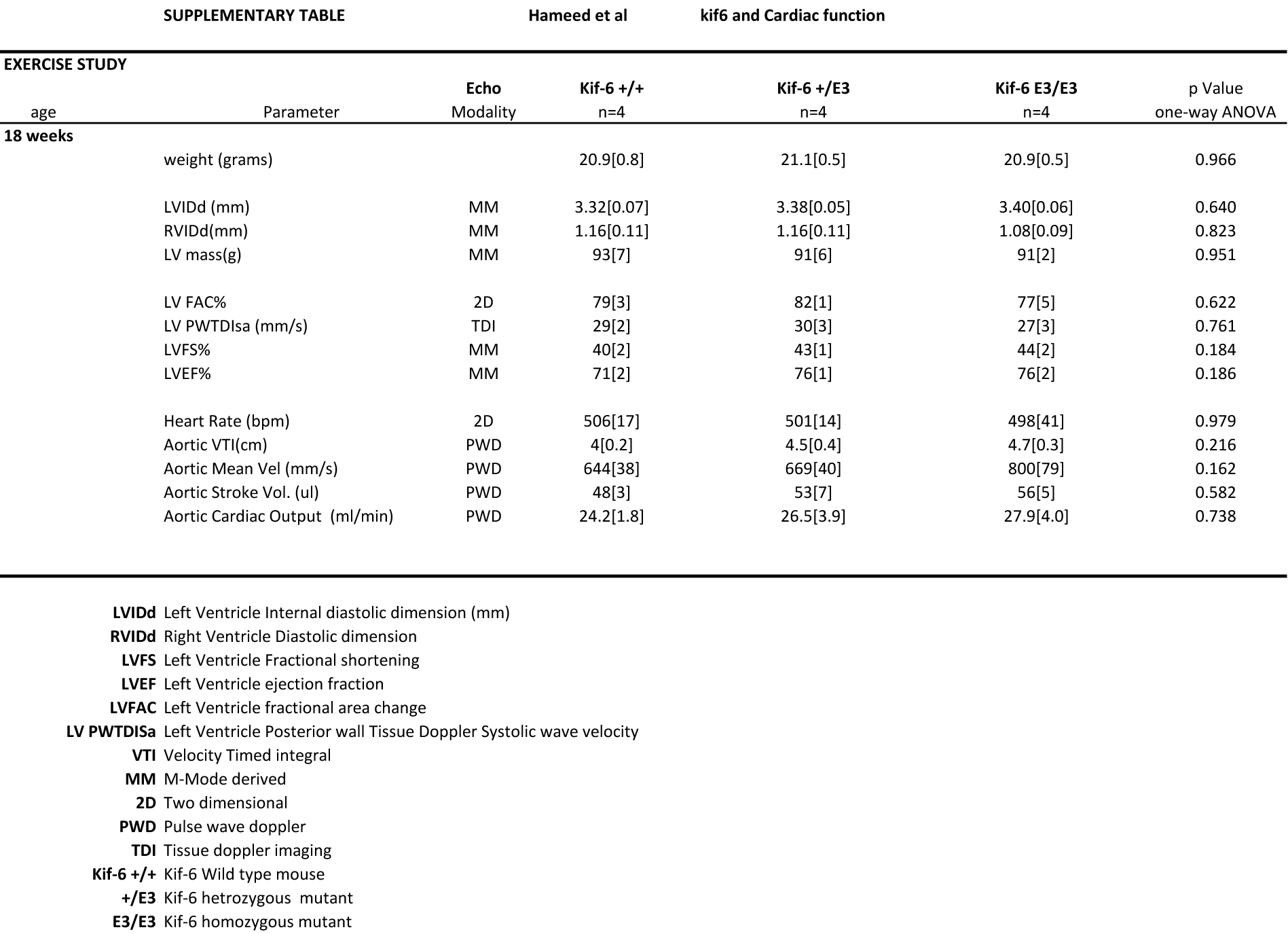

Supplement: Table S2 — Detailed physiological and echocardiographic dataset for adult female Kif6 mutant mice after continuous voluntary wheel running for 13 weeks. Data are presented as mean [SEM]. (TIF) [file pone.0054636.s004.tif]
