# Supplementary material for: No Evidence for Cardiac Dysfunction in Kif6 Mutant Mice
Source: PLoS One. 2013 Jan 23;8(1):e54636. doi: 10.1371/journal.pone.0054636 (PMC3552957; doi:10.1371/journal.pone.0054636)
Supplement: Table S1 — Detailed physiological and echocardiographic dataset for adult male Kif6 mutant mice undergoing serial echocardiography between 6–43 weeks of age. Data are presented as mean [SEM]. (PDF) [file pone.0054636.s003.pdf]

SUPPLEMENTARY TABLE 1 Hameed et al kif6 and Cardiac function

Age	Parameter	Echo Modality	C Kif-6 +/+ n=4	B Kif-6 +/E3 n=4	A Kif-6 E3/E3 n=3-4	p Value one-way ANOVA	
**6 weeks**							
	Body weight (grams)		20.3[0.5]	21.5[0.4]	20.2 [0.4]	0.114	
	LVIDd (mm)	MM	3.5[0.17]	3.6[0.03]	3.4[0.1]	0.066	
	RVIDd(mm)	MM	1.06[0.1]	1.11[0.08]	1.04[0.08]	0.869	
	LV mass(g)	MM	88[10]	89[5]	82[6]	0.761	
	LV FAC%	2D	76[3]	80[2]	79[4]	0.693	
	LV PWTDIsa (mm/s)	TDI	26[4]	31[1]	27[2]	0.342	
	LVFS%	MM	50[2]	44[2] *	52[1]	**0.020**	* P<0.05 Het vs. Hom
	LVEF%	MM	82[2]	76[2]*	84[1]	**0.018**	
	Heart Rate (bpm)	2D	490[28]	524[11]	500[19]	0.511	
	Aortic VTI(cm)	PWD	4.6[0.7]	4.3[0.4]	4.9[0.7]	0.794	
	Aortic Mean Vel (mm/s)	PWD	744[99]	711[85]	774[88]	0.889	
	Aortic Stroke Vol. (ul)	PWD	46[6]	42[4]	46[6]	0.857	
	Aortic Cardiac Output (ml/min)	PWD	23[4]	22[2]	23[3]	0.974	
**12 weeks**							
	Body weight (grams)		26.6[1.7]	26.2[0.8]	24.9[0.2]	0.639	
	LVIDd (mm)	MM	3.5[0.1]	3.4[0.1]	3.7[0.2]	0.449	
	RVIDd(mm)	MM	1.39[0.12]	1.24[0.1]	1.38[0.13]	0.593	
	LV mass(g)	MM	93[7]	95[11]	81[4]	0.522	
	LV FAC%	2D	69[5]	68[6]	67[5]	0.964	
	LV PWTDIsa (mm/s)	TDI	24[1]	31[2]	28[2]	0.089	
	LVFS%	MM	44[5]	41[4]	45[1]	0.764	
	LVEF%	MM	75[5]	72[6]	77[2]	0.773	
	Heart Rate (bpm)	2D	493[15]	532[14]	488[10]	0.105	
	Aortic VTI(cm)	PWD	4.7[0.2]	4.7[0.6]	4.8[0.1]	0.973	
	Aortic Mean Vel (mm/s)	PWD	783[37]	796[103]	786[60]	0.992	
	Aortic Stroke Vol. (ul)	PWD	48[4]	54[5]	50[4]	0.659	
	Aortic Cardiac Output (ml/min)	PWD	24[3]	28[3]	24[2]	0.385	
**18 weeks**							
	Body weight (grams)		30.4[1.9]	28.5[1.2]	27.8[0.4]	0.466	
	LVIDd (mm)	MM	3.55[0.09]	3.75[0.12]	3.78[0.05]	0.240	
	RVIDd(mm)	MM	1.32[0.12]	1.1[0.06]	1.24[0.02]	0.224	
	LV mass(g)	MM	91[8]	103[5]	90[4]	0.313	
	LV FAC%	2D	79[5]	75[3]	78[4]	0.798	
	LV PWTDIsa (mm/s)	TDI	28.8[2.8]	35.3[1.8]	24.3[2.9]	**0.047**	p=NS between groups
	LVFS%	MM	48[3]	37[2]	47[8]	0.199	
	LVEF%	MM	80[3]	67[3]	77[8]	0.167	
	Heart Rate (bpm)	2D	469[17]	503[16]	507[21]	0.269	
	Aortic VTI(cm)	PWD	4.6[0.6]	4.9[0.3]	5.3[0.6]	0.615	
	Aortic Mean Vel (mm/s)	PWD	756[118]	837[83]	873[103]	0.726	
	Aortic Stroke Vol. (ul)	PWD	54[6]	60[5]	62[9]	0.696	
	Aortic Cardiac Output (ml/min)	PWD	26[4]	30[3]	32[6]	0.574	
**43 weeks**			**n=2-3**		**n=2**	**t-test**	
	Body weight (grams)		39.8[0.52]		37.6[3.1]	0.434	
	LVIDd (mm)	MM	4.14[0.02]		3.87[0.14]	**0.088**	
	RVIDd(mm)	MM					
	LV mass(g)	MM	119[15]		122[16]	0.904	
	LV FAC%	2D	61[4]		65[2]	0.838	
	LV PWTDIsa (mm/s)	TDI	23.2[1.1]		23.3[0.1]	0.990	
	LVFS%	MM	34[1]		32[1]	0.251	
	LVEF%	MM	64[2]		60[2]	0.282	
	Heart Rate (bpm)	2D	465[4]		469[8]	0.630	
	Aortic VTI(cm)	PWD	5.6[0.3]		5.9[0.6]	0.419	
	Aortic Mean Vel (mm/s)	PWD	906[47]		1004[97]	0.332	
	Aortic Stroke Vol. (ul)	PWD	67[3]		78[9]	0.362	
	Aortic Cardiac Output (ml/min)	PWD	31[2]		37[5]	0.404	

LVIDd Left Ventricle Internal diastolic dimension (mm)
RVIDd Right Ventricle Diastolic dimension
LVFS Left Ventricle Fractional shortening
LVEF Left Ventricle ejection fraction
LVFAC Left Ventricle fractional area change
LV PWTDISa Left Ventricle Posterior wall Tissue Doppler Systolic wave velocity
VTI Velocity Timed integral
MM M-Mode derived
2D Two dimensional
PWD Pulse wave doppler
TDI Tissue doppler imaging
Kif-6 +/+ Kif-6 Wild type mouse
+/E3 Kif-6 hetrozygous mutant
E3/E3 Kif-6 homozygous mutant
