# Supplementary material for: No Evidence for Cardiac Dysfunction in Kif6 Mutant Mice
Source: PLoS One. 2013 Jan 23;8(1):e54636. doi: 10.1371/journal.pone.0054636 (PMC3552957; doi:10.1371/journal.pone.0054636)
Supplement: Methods S1 — Supplementary methods. (DOCX) [file pone.0054636.s005.docx]

**Supplementary methods**

***Mice***

Mice bearing an ENU-induced exon 3 splice acceptor site mutation in *KIF6* were identified by the RIKEN BioResource Center (Tsukuba, Japan). These mice were backcrossed to C57BL6/J mice for 5 generations.

Adult age and sex-matched mice were studied with food and water provided *ad libitum*. Male mice underwent serial echocardiography at age 6, 12 and 18 weeks. In a separate study, female mice were provided with a home cage running wheel [1] allowed to exercise from age 5 weeks, and underwent echocardiographic assessment at 18 weeks of age. Investigations were carried out in accordance with the Institution Board approval and licensed (40/3307) with the UK Home Office.

**Expression studies**

A mouse tissue blot was generated in house and Western Blotting performed as per standard protocols [2]. A c-myc epitope tagged version of Kif6a was expressed in HEK293 cells. Human umbilical vein endothelial cells were isolated from umbilical cords as described previously [3]. Ethical permission for use of umbilical cords was given by Institutional Board approval 10/H1308/25.

***Homology modeling***

We have used the SWISS-MODEL protein homology-modeling server to generate a three-dimensional homology model of the wild-type Kif6 motor domain [4]. The model was generated using the automated mode and the crystal structure of the human Kif9 motor domain in complex with ADP (PDB 3nwn), as a template.

**Lipid analysis**

Blood was collected via cardiac puncture into a desuridin-containing syringe (1:100 in blood) and serum or plasma separated by centrifugation. Triglyceride and cholesterol levels were measured at the Department of Clinical Chemistry (Royal Hallamshire Hospital, Sheffield, UK) using Roche Cobas® 8000 modular analyzer series.

***Echocardiography***

Transthoracic echocardiography was performed with a preclinical high frequency ultrasound imaging system (Vevo 770®, Visual Sonics, Toronto, Canada) using the RMV707B scan head. Mice were anaesthetised with isoflurane via oxygen before being placed supine on a heated platform and covered to minimise heat loss. Maintenance Isoflurane (0.5-1.5%) with oxygen was delivered via a nose cone and adjusted to achieve a heart rate near 500bpm, which was continuously recorded along with the respiration rate and rectal temperature. The chest of the mouse was depilated and preheated ultrasound gel was applied (Aquasonics 100 Gel, Parker Labs Inc. New Jersey, U.S) to allow subsequent image acquisition.

Standard parameters of the left ventricle were measured in the short axis view at the mid-papillary muscle level. Manual tracing of the LV end-diastolic and systolic areas were made to derive the fractional area change (FAC) as the primary index of contractility. M-Mode measurements were made for the LV wall and cavity dimensions (LVIDd), from which the ejection fraction (EF%), fractional shortening (FS%) and corrected LV mass were determined by standard automated analysis. Pulse wave tissue doppler (TDI) systolic wave (Sa) velocity was manually recorded from the endocardial aspect of the posterior wall of the left ventricle and represented another measure of contractility. Stroke volume was derived from measuring the velocity timed integral (VTi) of flow and diameter at the level of aortic valve annulus and multiplied by heart rate to obtain the cardiac output. From the right parasternal long axis view, right ventricle free wall measurements were recorded with M-Mode function. Mice were allowed to recover after each study.

Analysis was performed offline using the accompanying software (Vevo 770, V3.0). Measurements were taken during the relevant phase of the cardiac cycle that did not coincide with inspiration artefact. To minimise inter-observer variability all image acquisition and analyses were performed by a single, experienced operator (AGH) blind to the genotype of all subjects.

Continuous data are reported as mean [SEM]. All echocardiographic parameters were compared between the 3 groups of strains by one-way ANOVA for each time point with a Bonferroni post hoc test as indicated.

A p value of <0.05 was used to define statistical significance. Graphical and statistical analyses were performed with PRISM^®^ v.5.04 software (GraphPad Software, Inc, US).

1. De Bono JP, Adlam D, Paterson DJ, Channon KM (2006) Novel quantitative phenotypes of exercise training in mouse models. Am J Physiol Regul Integr Comp Physiol 290: R926-934.

2. Kasher PR, De Vos KJ, Wharton SB, Manser C, Bennett EJ, et al. (2009) Direct evidence for axonal transport defects in a novel mouse model of mutant spastin-induced hereditary spastic paraplegia (HSP) and human HSP patients. Journal of Neurochemistry 110: 34-44.

3. King AR, Francis SE, Bridgeman CJ, Bird H, Whyte MKB, Crossman DC. (2003) A role for caspase-1 in serum withdrawal apoptosis of human endothelial cells. Laboratory Investigation 83:1497-1508.

4. Arnold K, Bordoli L, Kopp J, Schwede T (2006) The SWISS-MODEL workspace: a web-based environment for protein structure homology modelling. Bioinformatics 22: 195-201.
